# Transcutaneous Electrical Nerve Stimulation Improves Stair Climbing Capacity in People with Knee Osteoarthritis

**DOI:** 10.1038/s41598-020-64176-0

**Published:** 2020-04-29

**Authors:** Hirotaka Iijima, Ryo Eguchi, Kanako Shimoura, Keisuke Yamada, Tomoki Aoyama, Masaki Takahashi

**Affiliations:** 1 Department of System Design Engineering, Faculty of Science and Technology, Keio University, Yokohama, Japan; 2 Department of Physical Therapy, Human Health Sciences, Graduate School of Medicine, Kyoto University, Kyoto, Japan; 3 Japan Society for the Promotion of Science, Tokyo, Japan; 4 School of Science for Open and Environmental Systems, Graduate School of Science and Technology, Keio University, Yokohama, Japan; 5 Development Center Technology Development HQ, Clinical Development Department, Omron Healthcare Co., Ltd, Kyoto, Japan

**Keywords:** Pain management, Rehabilitation

## Abstract

This study aimed to examine the effect of transcutaneous electrical nerve stimulation (TENS) on stair climbing capacity in individuals with pre-radiographic to mild knee osteoarthritis (OA). This is a secondary analysis of data from a single, participant-blinded, randomized controlled trial with a pre-post design. Participants with pre-radiographic to mild knee OA (mean age, 59.1 years; 72.9% women) were randomly assigned into two groups, a TENS (n = 30) and a sham-TENS groups (n = 29). TENS or sham-TENS treatments were applied to all participants by using the prototype TENS device with pre-specified parameters. The primary outcome measures included valid and reliable functional measures for stair climbing (stair-climb test [SCT]), visual analog scale for knee pain during the SCT, and quadriceps muscle strength. TENS improved SCT time by 0.41 s (95% confidence interval [CI]: 0.07, 0.75). The time reduction in the transition phase explains the TENS therapeutic effect. Post-hoc correlation analyses revealed a non-significant but positive relationship between the pain relief effect and improved 11-step SCT time in the TENS group but not in the sham-TENS group. These results indicate that the TENS intervention may be an option for reducing the burden of early-stage knee OA.

## Introduction

Knee osteoarthritis (OA) is a leading cause of knee pain and chronic disability in the elderly and subsequent mobility limitation^1,2^. Mobility limitation is typically considered one of the most significant consequences in individuals with knee OA. Weight-bearing activities that involve large knee flexion, such as stair climbing, are biomechanically and physiologically more challenging than level walking^3,4^ and are thought to be the first mobility limitation observed in individuals with knee OA^5^. A meta-analysis of 12 articles revealed that longer performance time was an alteration associated with knee OA during stair ambulation^6^. Although the mechanism of poor stair climbing capacity in knee OA is unclear, severe knee pain and consequent weaker quadriceps muscle power are potential modifiable factors that contribute to poor stair climbing capacity^7^. Pain-relieving intra-articular knee injection^8^ and quadriceps strength exercise^9^ improve stair climbing capacity, which indicates that treatment targeting these factors may alleviate the burden of the earliest stage of knee OA.

Transcutaneous electrical nerve stimulation (TENS) is an effective pain control modality for OA-related knee pain^10^. TENS treatment is beneficial for increasing isometric quadriceps muscle activation, possibly through reduced knee pain and increased quadriceps motor neuron pool excitability^11^, in patients with knee OA^12,13^. These evidences indicate that TENS may improve stair climbing capacity by reducing knee pain and increasing quadriceps muscle strength. Cherian *et al*. showed that 12 weeks of TENS treatment for individuals with severe knee OA did not significantly improve their stair climbing capacity^14^, which was possibly due to severe radiographic disease. Given that patients with less severe radiographic disease respond better to therapeutic interventions than those with severe disease^15,16^, TENS intervention targeting patients with less severe knee OA would be more effective in improving stair climbing function.

From a biomechanical perspective, stair descending involves higher knee extension moment, a leading cause of knee pain, than stair ascending^3,17^. The stair-climb test (SCT) is a validated and typical functional assessment for stair ambulation^18^ and can identify functional limitation better than other objectively measured functional assessments (e.g., timed up and go test and 6-minute walk test) in patients with knee OA^19^. However, the SCT incorporates stair ascending and descending, and their transition, which may represent distinct functional constructs. The total timed score of SCT limits our interpretation of the proportional contribution of these subtasks and therapeutic efficacy of the TENS treatment. As more severe pain and pain-related inhibition of muscle contraction were expected in stair descending, it was plausible that TENS treatment had a greater impact on stair descending than stair ascending. Thus, this study aimed to test the following hypotheses: (1) TENS has a greater impact on stair climbing capacity, the time to perform stair ascending, descending, and their transition, than sham TENS; (2) TENS is more effective for improving stair descending than ascending; (3) the beneficial effects of TENS on stair climbing capacity are driven through reduced knee pain; and (4) the beneficial effects of TENS on stair climbing capacity are driven through increased quadriceps muscle strength/activation in individuals with pre-radiographic to mild knee OA.

## Results

Fifty-nine participants (mean age, 59.1 years; mean body mass index [BMI]: 22.7 kg/m^2^; 72.9% were women) were included and randomized into the sham-TENS (n = 29) and TENS groups (n = 30). All the participants completed the post-treatment outcome measurements without any adverse events. Data from the results of the insole-based 11-step SCT (11-SCT) in the baseline period from one patient were imputed. Table 1 shows the comparison of participant characteristics between the sham-TENS and TENS groups.

**Table 1:** Comparison of baseline characteristics of the participants between individuals in sham-TENS (n = 29) and TENS (n = 30) groups.

Variable	Sham-TENS (n = 29)	TENS (n = 30)
Age, years	58.2 ± 5.63	59.9 ± 6.41
Female, no. (%)	20 (69.0)	23 (76.7)
Height, m	1.60 ± 0.08	1.61 ± 0.08
Mass, kg	60.2 ± 12.6	57.2 ± 8.00
BMI, kg/m^2^	23.4 ± 4.03	22.1 ± 2.94
Index knee K&L grade, no. (%)		
Grade 0	12 (41.4)	11 (36.7)
Grade 1	13 (44.8)	14 (46.7)
Grade 2	4 (13.8)	5 (16.7)
JKOM, points†		
Pain and stiffness	6.07 ± 3.29; 6 [0, 15]*	7.90 ± 4.25; 7 [2, 22]*
Activities of daily living	2.48 ± 3.10; 2 [0, 13]*	3.47 ± 3.05; 3 [0, 14]*
Participation in social activities	2.07 ± 1.62; 2 [0, 6]*	2.80 ± 2.19; 2 [0, 9]*
General health conditions	1.93 ± 0.84; 2 [0, 3]*	2.00 ± 1.11; 2 [0, 4]*
Total score	12.6 ± 6.29; 12 [3, 32]*	16.2 ± 8.40; 14 [5, 49]*

BMI, body mass index; JKOM, Japanese Knee Osteoarthritis Measure; K&L grade, Kellgren and Lawrence grade.

Values are presented as mean ± SD except where otherwise indicated.

*Median [lower range, upper range] was also provided because of the scattered distribution of the answered items.

^†^Higher JKOM scores indicate worse status.

Table 2 shows a comparison of changes in outcome measures between the sham-TENS and TENS groups after multiple imputation. The between-group differences in change in knee pain and quadriceps muscle strength were not significantly different. The proportion of treatment responders (i.e., visual analog scale [VAS] pain score improved by ≥30% from baseline to post-treatment) did not differ between the sham-TENS and TENS groups (relative risk to responder in the TENS group: 1.03; 95% confidence interval [CI]: 0.65, 1.62). Although the between-group difference in the change in stopwatch-based 11-SCT time was not significant, that in the insole-based 11-SCT time was 0.41 s (95% CI: − 0.75, −0.07 s), which showed better improvement in the patients in the TENS group than in those in the sham-TENS group. When insole-based 11-SCT time was further divided into three phases (ascending, descending, and transition), the between-group difference in the transition phase time was 0.32 s, which was more improved in patients in the TENS group than in those in the sham-TENS group. The case completed data, excluding one patient in the sham-TENS group, showed similar results (data not shown).

**Table 2:** Change within-group and difference in change between groups for VAS pain, quadriceps muscle strength, and stair climbing capacity.

Variable	Sham-TENS (n = 29)		TENS (n = 30)		Adjusted difference in meanat post-treatment period†
	Baseline	Post-treatment	Baseline	Post-treatment	
**Knee pain**					
VAS pain score in SCT, mm	16.4 ± 18.9	10.9 ± 14.0	15.1 ± 15.8	7.60 ± 12.9	−2.62 (−7.74, 2.48)
Quadriceps strength, Nm/kg	1.41 ± 0.43	1.53 ± 0.47	1.41 ± 0.57	1.45 ± 0.60	−0.08 (−0.26, 0.10)
**Stair climb capacity**					
11-SCT (stopwatch), s	8.76 ± 1.36	8.68 ± 1.41	9.32 ± 1.33	8.93 ± 1.14	−0.22 (−0.54, 0.10)
11-SCT (insole), s	**9.07** ± **1.37**	**9.10** ± **1.53**	**9.68** ± **1.43**	**9.23** ± **1.24**	**−0.41 (−0.75, −0.07)**
Ascending phase, s	4.67 ± 0.68	4.55 ± 0.67	4.87 ± 0.73	4.75 ± 0.56	0.07 (−0.16, 0.30)
Descending phase, s	4.07 ± 0.60	4.06 ± 0.62	4.40 ± 0.69	4.27 ± 0.67	−0.08 (−0.26, 0.10)
Transition phase, s	**0.34** ± **0.29**	**0.49** ± **0.49**	**0.40** ± **0.36**	**0.20** ± **0.39**	**−0.32 (−0.53, −0.10)**

VAS, visual analog scale; SCT, step stair climb test; 95% CI: confidence interval.

*In the sham-TENS group, the missing value of 11-SCT from one patient was imputed using multiple imputation technique.

^†^The mean (95% CI) between-group difference was calculated after adjusting for baseline value.

Bold type represents a statistically significant result.

**Figure 1:**
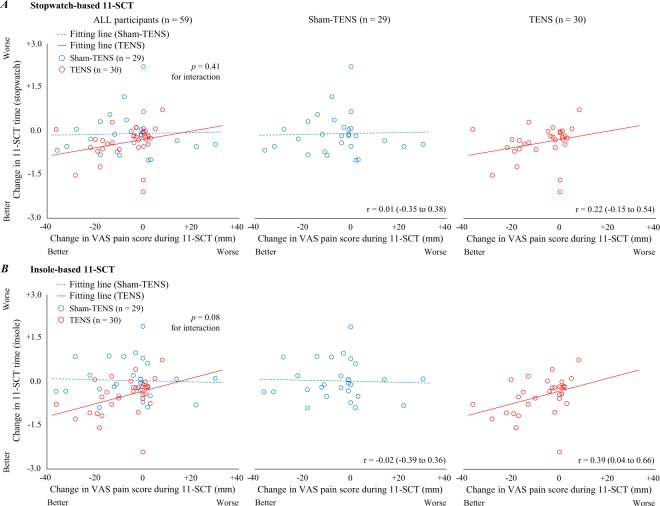
Illustration of the relationship between pain relief and therapeutic effect on stair climbing capacity. (**A)** Change in VAS pain score during SCT and change in stopwatch-based 11-SCT time. (**B)** Change in VAS pain score during 11-SCT and change in insole-based 11-SCT time. Linear regression lines and Pearson correlation coefficients (95% confidence intervals) are provided. *p*-values for group-change in VAS pain score interaction effect are also provided.

A graphic illustration of these relationships is provided in Fig. 1 to further address the relationship between the pain-relief and therapeutic effect of TENS on the stopwatch- (Fig. 1A) and insole-based 11 SCT times (Fig. 1B). The post-hoc correlation analyses revealed a positive relationship between the change in VAS pain score during SCT and the change in stopwatch- (*r* = 0.22; 95% CI: −0.15, 0.54) and insole-based 11-SCT times (*r* = 0.39; 95% CI: 0.04, 0.66) in the TENS group, which were higher than those in the sham-TENS group (stopwatch-based 11-SCT, *r* = 0.01; 95% CI: −0.35, 0.38; insole-based 11-SCT, *r* = −0.02; 95% CI: −0.39, 0.36). The interaction effect of group × change in VAS pain score on the insole-based 11-SCT time was marginally significant (*p* = 0.080).

## Discussion

**Figure 2:**
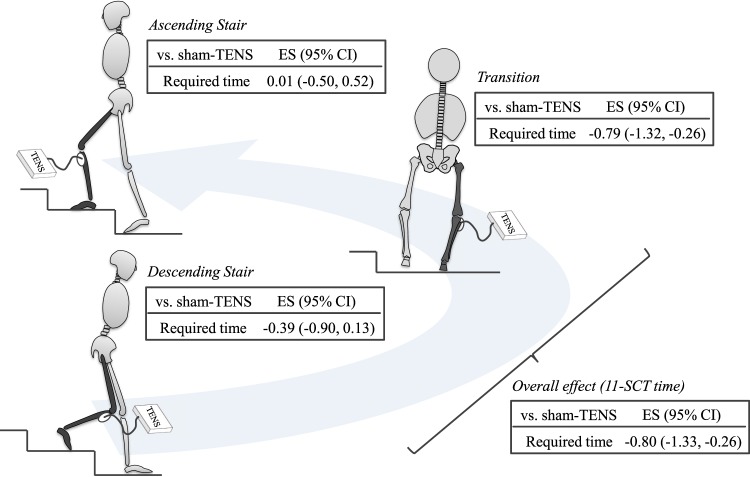
Graphic abstract. TENS improved insole-based 11-SCT time (ES: −0.80; 95% CI: −1.33, −0.26) better than sham-TENS in people with pre-radiographic to mild knee OA, which was through reducing transition time (ES: −0.79; 95% CI: −0.1.32, −0.26) rather than ascending (ES: 0.01; −0.50, 0.52) and descending stair time (ES: −0.39; −0.90, 0.13). ES, effect size; 95% CI, 95% confidence interval.

The objective of this study was to examine the therapeutic effects of TENS on stair climbing capacity in individuals with pre-radiographic to mild knee OA. Supporting the first hypothesis, TENS improved the 11-SCT time better than sham-TENS. However, contrary to the second hypothesis, the therapeutic effect of TENS was similar between stair descending and ascending during the 11-SCT. Furthermore, TENS was not superior to sham-TENS in improving knee pain during stair climbing and quadriceps strength, which does not support the third and fourth hypotheses. The improved stair climbing ability by TENS was not due to the reduced knee pain and increased quadriceps muscle strength, although the post-hoc correlation analyses revealed a non-significant but positive relationship between the pain relief effect and improved 11-SCT time in the TENS group. The main finding of this study is that TENS improved stair climbing capacity, determined using a sensor-mounted insole, better than sham-TENS in the patients with earlier radiographic OA stage. The time reduction in the transition phase during TENS treatment mostly explained the therapeutic effects (see graphic abstract, Fig. 2). As poor stair climbing capacity is thought to be the first mobility limitation observed in individuals with knee OA^5^, these findings indicate that TENS may be an option for reducing early-stage knee OA-related burden.

### Interpretation of the Effects of TENS on Stair Climbing Capacity

In this study, we found that TENS improved insole-based SCT time by 0.41 s, which is beyond the 95% CI of measurement error. This therapeutic effect on SCT time is not consistent with the findings of a previous study that showed that 12 weeks of TENS treatment in individuals with severe knee OA did not significantly improve SCT time^14^. This discordance may be attributed to the difference in radiographic disease severity. Approximately 85% of the study participants had pre-radiographic knee OA (K&L grade ≤1), who may respond better to therapeutic interventions than those with severe disease^15,16^. Furthermore, insole-based SCT may be more sensitive than stopwatch-based SCT in detecting the efficacy of therapeutic treatment, although the stopwatch-based SCT has high reliability and validity^18^. It should be acknowledged that this study is a secondary analysis from randomized controlled trial (RCT) data without calculation of the required sample size to detect the therapeutic effect of TENS on 11-SCT time. Thus, a type II error may exist, and the non-significant TENS treatment effects on the stopwatch-based 11-SCT time may be attributed to the low statistical power, given that the upper limit of the 95% CI in the stopwatch-based 11-SCT improvement was almost zero.

Contrary to the hypothesis, TENS did not significantly improve the time during stair descending that requires higher internal knee extension moment^3,17^. It is Interesting that the time reduction in the transition phase during TENS treatment mostly explained its therapeutic effects. Although the exact mechanism is unclear, the transition phase involves a quick turn that increases the external knee rotational moment^20^, which may cause knee pain and a subsequent impairment of smooth transition from stair descending to ascending. This preliminary finding warrants validation of the association between knee pain and impaired transition motion, and whether the transition motion responds well to pain-relief treatment.

Theoretically, the TENS treatment reduces pain through selective stimulation for large-diameter, low-threshold non-noxious afferents in dermatomes related to pain^21^ and increases quadriceps motor neuron pool excitability^11^. Nevertheless, this study could not detect significant between-group improvements in knee pain during 11-SCT and quadriceps muscle strength immediately after TENS, although the same TENS protocol improved knee pain and walk distance during 6-minute walk test^22^. Further studies would be needed to determine what factors mediate the therapeutic effect of TENS. As improved knee pain was associated with improved 11-SCT time only in the TENS group, the underlying mechanism associated with change in 11-SCT may be different between the sham-TENS and TENS groups.

### Significance and Clinical Impact of the Study

TENS was effective in improving mobility during insole-based 11-SCT, including people with pre-radiographic OA, the earliest clinical manifestation associated with knee OA^5^. This finding may help clinicians and physical therapists provide effective therapy for patients with early-knee OA who have difficulty performing weight-bearing activities that involve large knee flexion. TENS may be an option for reducing early-stage knee OA-related burden, which should be validated in future clinical trials.

The type of measurement metrics is important for identifying the therapeutic effects of TENS, and this study used both the stopwatch- and insole-based 11-step SCT. The stopwatch-based 11-SCT may not adequately identify the therapeutic effects of TENS, although non-significant results may come from the low sample size. Thus, biomechanically and physiologically more challenging tasks (e.g., 15-step SCT) might be suitable in adequately determining the therapeutic effect by using a stopwatch in the clinical setting. Further studies are warranted to address this question.

### Study limitations

First, this study examined the immediate effects of TENS without follow-up. This study cannot specify optimal TENS treatment protocol. Second, the knee pain intensity on the testing day was quite mild in both groups, although all the participants experienced pain at a mean score of >4 but <9 on a numeric rating scale. As severe knee pain at baseline could influence the pain relief effects of the TENS treatment, the relatively mild pain profile of the included participants may underestimate the pain relief effects of the TENS treatment. Third, the 11-SCT in this study was performed while wearing standard shoes, which may not be used in clinical practice. Data should be interpreted with caution because most clinicians would perform stopwatch-based 11-SCT while the participants wear their own footwear, which might influence the SCT time. Fourth, this study did not calculate the required sample size, as this study is a secondary analysis of RCT data^22^. Finally, the lack of muscle activity data such as electromyography data limited our analysis. As patients are required to use their quadriceps submaximally, the muscle activity during the 11-SCT for quadriceps motor neuron activation may be a better option for identifying the therapeutic effect of TENS.

In conclusion, TENS immediately improved the 11-SCT time better than sham-TENS. However, the therapeutic effect of TENS was similar between stair descending and ascending during the 11-SCT, and the time reduction in the transition phase during the TENS treatment mostly explained its therapeutic effects. The improved stair climbing ability by TENS was not due to the reduced knee pain and increased quadriceps muscle strength, although post-hoc correlation analyses revealed a non-significant positive relationship between the pain relief effect and the improved 11-SCT time in the TENS group. Given that stair climbing is suggested to be the first task that is affected in people with knee OA, TENS intervention may be an option for reducing early-stage knee OA-related burden, which should be validated in future clinical trials.

## Methods

### Study design and participants

This study was a secondary analysis of data from a RCT that examined the effects of TENS on knee pain and physical function in individuals with painful knee OA^22^. The institutional ethics committee of Kyoto University Graduate School and Faculty of Medicine approved the study (approval No. C1349). The study was conducted in compliance with Good Clinical Practice, the ethical standards of the responsible committee on human experimentation (institutional and national) and the Declaration of Helsinki 1975. Written informed consent was obtained from all participants before their enrollment. The required sample size in the RCT was calculated on the basis of the data from a previous clinical trial to detect the therapeutic effects of TENS. Details for sample size calculation were provided in the original RCT study^22^. Community-dwelling adults who reported knee pain within the last month were identified through a website. Subjects interested in this study answered a questionnaire concerning their physical condition, and those who met the inclusion criteria were invited to the university in February 2018 to participate in the study. The eligibility criteria included (1) age of ≥50 years; (2) Kellgren and Lawrence (K&L) grade of ≤2 in one or both knees as evaluated using weight-bearing anteroposterior radiographs, and (3) mean pain score of ≥4 but ≤9 on a numeric rating scale (0–10 total points) in the last month. The participants were excluded if they (1) had a history of knee surgery or intra-articular corticosteroid or hyaluronic acid injection within the last 6 months, (2) had a history of knee joint replacement or tibial osteotomy, (3) were receiving physical therapy, (4) had pain in any other major joints (e.g. back, hip, or ankle) with a greater effect than their knee pain on the current ability limits, (5) had severe medical conditions (e.g., chronic obstructive pulmonary disease, cardiovascular disease, arteriosclerosis obliterans, cerebrovascular accident, lumbar disk herniation, and rheumatoid arthritis), (6) did not usually use stairs in daily living, and (7) did not have the ability to walk and climb stairs without an ambulatory assistive device and handrail. Although the original RCT excluded patients with K&L grade 2 after data assessment^22^, this secondary study included these patients to increase its statistical power.

### Randomization

The participants were randomly allocated to one of the two groups, the TENS or sham-TENS group, by using stratified and block randomization^22^. Studies have shown that female patients have a lower sensory threshold and respond to electrical stimulation differently from male patients;^7,8^ thus, blocks were stratified by sex. Details for randomization were provided in the original RCT study^22^.

### TENS Intervention

The TENS and sham-TENS treatments were applied to index knee of all participants using the prototype TENS device (HV-F710, Omron Healthcare Co., Ltd., Kyoto, Japan) with pre-specified parameters (sweep mode from 1 to 250 Hz, symmetrical biphasic pulse, and a 60-μs pulse width). Sweep mode was chosen because low and high frequency TENS demonstrate analgesic effects in different mechanism, and the combination of these frequencies is supposed to be more effective in reducing pain than alone^23^. The index knee was defined as the more painful knee at inclusion. The intensity in the TENS group was gradually increased and limited by the participant’s perception of a “strong but comfortable, non-painful tingle” immediately below the visible motor threshold. The participants in the sham-TENS group received sham stimulation at the same sites. TENS and sham-TENS were applied just before functional assessments explained in original RCT study^22^. Treatment time was not strictly set because previous Cochrane review showed similar therapeutic effects on knee pain and functional outcomes regardless of treatment time of ≤20 min, 30 to 40 min, and 60 ≥ min^24^. The TENS units were turned off when all the functional assessments were completed, for a total application time of around 60 minutes. Trained physical therapists with clinical experience in the musculoskeletal field performed the procedures. More information on the treatment procedure was provided in the original RCT^22^.

### Outcome measurement

The participants were assessed by the same assessor at baseline and during the treatment period. The primary outcome measures included valid and reliable functional measures for stair climbing, VAS for knee pain during SCT, and quadriceps muscle strength.

### Instrumentation

Footwear influences balance performance^25,26^ and walking speed^26^; thus, all the participants wore standard shoes (LD AROUND M, Mizuno, Tokyo, Japan) with a pressure sensor (Force register sensors Model 402, Interlink Electronics Inc., CA, USA) to avoid any potential bias from the participants’ wearing of their own footwear^27^. Fifteen sensors were attached in the shoe insole to enable detection of the precise initial contact and foot off time point during the SCT. During stair climbing, forefoot contact without heel contact was expected, and a pilot study for healthy adults indeed confirmed the forefoot initial contact during stair descending (data not shown). In-shoe foot pressure sensors can more precisely detect foot contact than single foot sensor. Furthermore, this method allows examination of the stair climbing capacity using actual stairs outside the motion laboratory. The sensors were connected to a flexible circuit board containing a microcontroller and micro-SD card placed on the participants’ lower legs^27^. Although the footwear was novel for each participant, all the subjects reported that the shoes were comfortable and caused no pain. Data were sampled at 100 Hz.

### Procedure for the 11-Step Stair-Climb Test

Each participant underwent the SCT in accordance with the OARSI recommended method^28^, while wearing pressure sensor-mounted standard shoes. To exclude the influence of the participants’ clothes on the stopwatch operability, the participants’ pants were rolled up to the knee level. The 11-SCT with a regular stairwell was selected because it represents the type and size of stairs that subjects likely need to manage during daily activities, and some previous studies that examined reliability also used 11-SCT^29,30^. The participants descended and ascended a flight of stairs, which consisted of 11 steps with step height, width, and tread of 17, 135, and 29 cm, respectively. Although the standard SCT starts from the ascending stairs, the 11-SCT in this study started from descending stairs because of environmental constraints. The pilot study confirmed that the 11-SCT time in the 6 healthy adults (mean age: 24.3 years) did not significantly differ between starting ascending and descending (data not shown). A trained physical therapist (KS) measured the time required to perform 11-SCT in all the participants’ test and retest sessions by using a stopwatch (TD-392, Tanita Corp., Tokyo, Japan). On the trained physical therapist’s “start” command, each participant was instructed to walk as quickly and safely as possible without running^31^ and was subsequently retested to ensure that their physical condition was stable over the testing period. The use of any walking aid or handrail was prohibited. The participants performed one practice trial for familiarization and to verify their safety. Stopwatch-based 11-SCT time was defined as the time from the therapist’s command to “start” to the subject reaching the point where both feet were on the top platform. The stopwatch and insole measurements were performed simultaneously.

To determine insole-based 11-SCT time, vertical ground reaction force (GRF) was calculated from the sensor-mounted shoe data as previously described^27^. Subsequently, initial contact and foot off were defined as the time point where the vertical GRF exceeded and was <1% of the threshold of the estimated maximum GRF using MATLAB version R2017a (MathWorks Inc., Natick, MA, USA). The insole-based 11-SCT time was defined as the time from the initial foot off on the top platform to the point of foot contact in both feet on the top platform. By using the initial contact and foot off data, the insole-based 11-SCT time was further divided into three phases, namely descending, ascending, and transition. The transition phase was defined as the time between initial contact to staircase landing after descending and initial contact to the first step during ascending. The test–retest reliability was excellent for the stopwatch-based (intraclass correlation coefficient [ICC_1,1_], 0.952 s; 95% CI, 0.560, 0.985 s) and insole-based 11-SCT times (ICC_1,1_, 0.929 s; 95% CI, 0.883, 0.957 s). The minimal detectable changes (MDC_95_) for stopwatch- and insole-based 11-SCT times were 0.102 s and 0.089 s, respectively.

### Knee Pain Intensity during Stair-Climb Test

Knee pain during SCT was evaluated after the 11-SCT by using the VAS (0–100 mm). Each participant was asked the following “Please mark on where you think the level of pain you experienced during stair climbing.” The participants recorded their level of knee pain by drawing a vertical mark between the ends of a 100-mm horizontal line. The 0- and the 100-mm end represented no pain and most extreme pain, respectively. The distance from the 0-mm end to the drawn mark was recorded to the first decimal place.

### Quadriceps muscle strength

The maximum isometric quadriceps (Nm/kg) in both legs were measured using a hand-held dynamometer (HHD; μTas F-1, Anima Corp., Tokyo, Japan) in accordance with a method previously validated for use on community-dwelling elderly fallers^32^. The participants were instructed to remain seated in an upright position. The knee was placed in 90° flexion, with the HHD attached 10 cm proximal to the lateral malleolus and held in place with an inelastic strap that was looped around the therapy bed and fastened. The length of the straps allowed for an isometric contraction to be performed, with the knee at 90° flexion during testing. The participants were instructed to extend their leg for 5 s. Strong verbal encouragement was provided to ensure maximum effort. To provide moment value (Nm), the lever arm (length of tibia) between the knee joint and the HHD was manually measured and subsequently normalized to their mass (Nm/kg). The means of the values from the two exercises were used for statistical analysis.

The MDC_95_ was calculated using 100 randomly selected participants (i.e., 200 knees) to determine the smallest degree of change outside the muscle strength testing error range. The MDC_95_ was 0.227 Nm/kg. The intra-rater reliability was excellent for quadriceps strength (ICC_1,1_: 0.939; 95% CI: 0.921, 0.954).

### Patient Characteristics

Data on age, sex, and height were self-reported by the patients. Body mass was measured on a stadiometer with the participants clothed without shoes. BMI was calculated by dividing the weight in kilometers by the height in meters squared. The knee OA-related health domain measure, the Japanese Knee Osteoarthritis Measure (JKOM), was evaluated^33^. The concurrent and construct validities of the JKOM were established by comparing the Western Ontario and McMaster Universities Arthritis Index and the Medical Outcomes Study 36-item Short-Form Health Survey score^33^. Radiographic OA severity in both knees was assessed in the anteroposterior short view in the weight-bearing position, similarly to a previous study^34^.

### Statistical analyses

Statistical analysis was performed on an intention-to-treat basis and included all patients who were randomized into two groups. To account for missing data, primary analyses included multiple imputation technique. Imputation of insole-based 11-SCT for one patient data was conducted. Imputed data set was formed using the multiple imputation procedure of SPSS Statistics for Windows, Version 23.0 (IBM Corp., NY, USA). Sensitivity analysis was performed using all available data without imputation (complete cases).

Multiple regression analysis was performed with TENS intervention (0: no, 1: yes) as independent variable and with each outcome measure (continuous) as dependent variable after adjusting for the baseline value of each outcome. The change in VAS pain score during SCT was dichotomized as “responder” (the VAS pain score improved by ≥30% from baseline to post-treatment^35^) and “non-responder.” Between-group comparisons were performed using log binomial regression and presented as relative risks.

Post-hoc correlation analysis was performed to address the relationship between the pain relief and therapeutic effects of TENS on SCT time stratified by group (sham-TENS and TENS). In this analysis, the Pearson correlation coefficients between the change in VAS pain score and 11-SCT time were evaluated in each group. The interaction effect of group × change in VAS pain score on change in 11-SCT time was also evaluated. Data analyses were performed using JMP Pro 13.0 (SAS Institute, Cary, NC, USA). Statistical significance was determined with *p* < 0.05.

## Data Availability

The datasets generated and/or analyzed in the present study are available from the corresponding author on reasonable request.
